# Development of a community-based hearing loss prevention and control service model in Guangdong, China

**DOI:** 10.1186/s12889-019-7910-y

**Published:** 2019-11-29

**Authors:** Chang Liu, Anshi Wang, Yanlin Huang, Yan Zhang, Hongke Ding, Jing Wu, Li Du, Jie Yang, Fei Mai, Yukun Zeng, Ling Liu, Xin Zhao, Changbin Zhang, Aihua Yin

**Affiliations:** 1 Medical Genetic Center, Guangdong Women and Children Hospital, Guangzhou, 510010 Guangdong China; 2 Maternal and Children Metabolic-Genetic Key Laboratory, Guangdong Women and Children Hospital, Guangzhou, 510010 Guangdong China; 3 Department of Neonatolog, Guangdong Women and Children Hospital, Guangzhou, 510010 Guangdong China; 4 Department of ENT, Guangdong Women and Children Hospital, Guangzhou, 510010 Guangdong China

**Keywords:** Hearing loss, Prevention and control, Service model, Clinical program

## Abstract

**Background:**

Hearing loss is a prevalent sensorineural disorder and a major public health issue in China. It is suggested that half of all cases of hearing loss can be prevented through public health measures. However, national strategies for hearing healthcare are not implemented well in Guangdong and some other regions in China.

**Methods:**

To develop a community-based service model for the prevention and control of hearing loss in Guangdong, we integrated the model with multiple maternal and child healthcare models, and set up a series of clinical programs along with an optimum timeline for the preventive measures and intervention treatments to take place. A total of 36,090 families were enrolled in the study, including 358 high-risk families and 35,732 general-risk families.

**Results:**

The study lasted for 6.5 years, and 30,769 children were born during that period. A total of 42 children were born with congenital deafness; 17 of them were born into families with advanced genetic risks for hearing loss, 9 were born with specific medical conditions, and 16 were born into general-risk families. About one third of them were diagnosed prenatally, others were diagnosed within 3 months of age, and 72% of them received interventions initiated before 6 months of age. 13 children presented with delayed hearing loss; 9 of them were diagnosed with delayed hereditary sensorineural deafness in neonatal period, and 4 were diagnosed within 3 months after onset. Timely interventions were provided to them, with appropriate referrals and follow-ups. Beside these, 80 families were identified with genetic susceptibility to aminoglycoside ototoxicity. Detailed medication guides were provided to prevent aminoglycoside-induced hearing loss. Moreover, through health education and risk reduction strategies, the prevalence of TORCH syndrome decreased from 10.7 to 5.2 per 10,000. Additionaly, the awareness rates of health knowledge about hearing healthcare significantly increased in the cohort.

**Conclusions:**

Adapting national strategies for local or district projects could be an important step in implementing hearing loss prevention measures, and developing community-based service models could be of importance in carrying them out.

## Background

Hearing loss is a prevalent sensorineural disorder and a major public health issue, associated with increased cognitive obstacles, poorer social functioning, and increased health care costs [1–3]. According to the World Health Organization’s estimates, approximately 466 million people worldwide suffer from disabling hearing loss, and 34 million of those are children [4]. In China, 27.8 million people have disabling hearing loss, accounting for 33.5% of the total registered disabled [5]. With the world’s largest population and the new family-planning policies, hearing loss has become a significant public health concern in China [5–7].

Hearing loss may result from genetic factors, infection factors, perinatal or neonatal complications, ototoxic drugs, excessive noise and other causes [8]. It is suggested that over half of all cases of hearing loss can be prevented through public health measures [9, 10]. However, the national strategies for hearing healthcare are not implemented well in Guangdong and some other regions in China. Establishing regional service models could be of importance for the implemention of national strategies to prevent and treat hearing loss. In China, the maternal and child health care has solid foundation, and its service models are inclusive. To develop a community-based service model for the prevention and control of hearing loss in Guangdong, we integrated the model with multiple maternal and child healthcare models, and set up a series of clinical programs along with an optimum timeline for the preventive measures and intervention treatments to take place.

## Methods

### Subjects

The study was approved by Ethics Committee of Guangdong Women and Children Hospital of Guangzhou Medical University. A total of 36,090 families were enrolled in the study under an institutional review board-approved protocol. The cohort included 358 high-risk families (with hearing impairments or family histories of hearing loss), as well as 35,732 general-risk families (with normal hearing and without family history of hearing loss). The study cohort was recruited from October 2010 to February 2017. All participants have given their written informed consents. All the procedures performed in the study were in accordance with the Declaration of Helsinki.

## Develop a service model consisting of prevention programs at different life stages

### Prevention of hearing loss in pre-conception stage

During pre-conception counseling, medical records for each participating families were obtained, including clinical histories, infection histories, histories of abnormal pregnancies, use of ototoxic drugs, occupational exposures, as well as genetic factors related to hearing loss. For families with hearing impairments or family histories of hearing loss, clinical evaluation and genetic testing were recommended. As to hearing families without family history of hearing loss, health education and screening tests were provided. For couples identified with pathogenic mutations, recurrence information and reproductive choices were provided. Moreover, TORCH (Toxoplasmosis, Other: syphilis, varicella-zoster, Rubella, Cytomegalovirus, and Herpes infections) complex screening was recommended to pre-pregnant women for the prevention and control of toxoplasmosis, rubella, cytomegalovirus, herpes simplex virus, HIV, syphilis and other common congenital infections. Effective vaccines are available for many of the viruses that cause hearing loss, and may lead to substantial changes in the incidence of these infections in the offsprings of these pre-pregnant women. Interventions for the prevention and treatment were undertaken according to the guidelines [11]. Additionally, health education and risk reduction strategies were provided through harm-reduction counseling. Nutritional advice and vitamins supplementation were given to prevent fetal malnutrition and associated deafness [12, 13].

### Prevention of hearing loss in pregnancy stage

Clinical evaluation and genetic testing were provided to 358 high-risk families through the diagnosis program of hearing loss. Physical examination and screening tests were provided to 35,732 general-risk families through a screening program. In the screening program of hearing loss, allele-specific PCR-based universal array was applied to screen hotspot mutation carriers of deafness-associated genes. In the diagnosis program, a microarray assay was used to initially select hotspot mutations of genes associated with hereditary hearing loss, and for those with hearing impairment but tested negative for hotspot mutations, DNA sequence analysis were performed. Details about both the programs were described in our previous reports [14, 15]. The carrier couples identified during pregnancy may choose prenatal diagnosis and get prepared for the health and educational needs of affected neonates. Moreover, risk reduction strategies were provided to those who showed advanced risks in TORCH complex screening, and the therapeutic effects were monitored. Further examination and proper management were conducted to control important causes of congenital anomalies and associated hearing impairment [16].

### Prevention of hearing loss in perinatal stage

Improve birth practice, and try to avoid birth trauma and hypoxia. For high-risk participants with HSV lesion or prodrome presented at the time of delivery, cesarean section was recommended according to guidelines to avoid vertically transmitted infection [17]. For neonates with specific medical conditions, such as prematurity and (or) low birth weight, low Apgar scores, NICU stay, hyperbilirubinaemia, as well as in the use of ventilation, close attention and audiological assessment were provided to avoid perinatal complications and associated hearing impairment. For neonates who needed drugs such as aminoglycoside antibiotics, drug sensitivity and genetic susceptibility were tested to obtain good therapeutic effect and to avoid drug-induced hearing loss.

### Prevention of hearing loss in neonatal period

Universal newborn hearing screening (UNHS) was carried out for infants, using the transient evoked otoacoustic emissions and auditory evoked potentials as the first two steps, and automated auditory brainstem response (ABR) test as the third step. As to cases with increased genetic risks, genetic testing was also recommended. Moreover, we screened for G6PD deficiency, blood group incompatibility and other risk factors. In those newborns with advanced risks of hemolytic diseases, timely measures were taken to avoid jaundice caused auditory neuropathy and hearing loss. In addition, avoidance of ototoxic drugs and reduction in exposure to excessive noise were recommended for the prevention of hearing impairment.

### Prevention of hearing loss in childhood and adolescence

In childhood and adolescence, health education is important in promoting personal hygiene and avoiding impacted cerumen, as well as in preventing otitis externa. Better management of upper respiratory tract infections help reduce otitis media. Immunization and prophylaxis help avoid measles, mumps and meningitis. Thus, for children and adolescents, we developed a student hearing healthcare program to promote personal hygiene and ear care, to help them better manage upper respiratory tract infections, to promote immunization against illnesses related to hearing loss, and to increase their awareness about harmful factors. We gave health education in schools and communities, organized health knowledge quizzes and other fun activities to increase their awareness of harmful factors and preventive measures.

### Prevention of hearing loss in adulthood

To promote a positive image of hearing, we developed a family hearing healthcare program in our ear center and brought it to the communities. We gave health care consultations in the chinic, provided health education in the communities, hosted workshops and distributed brochures. Of those, excessive noise is a major avoidable cause of hearing impairment. In China, occupational noise and urban, environmental noise are significant risk factors for hearing impairment. Because there is widespread ignorance of the hazard, awareness should be increased about the harmful effects of noise on hearing and about the prevention and control of noise-induced hearing loss. Thus, in adults, protection against excessive and/or prolonged noise exposure, as well as ototoxic solvents and industrial chemicals were provided. Moreover, a surveillance system and a self-reporting system were developed in the program, so that timely interventions and appropriate referrals could be provided.

## Results

### Implementation of prevention and control programs in the service model

**Fig. 1:**
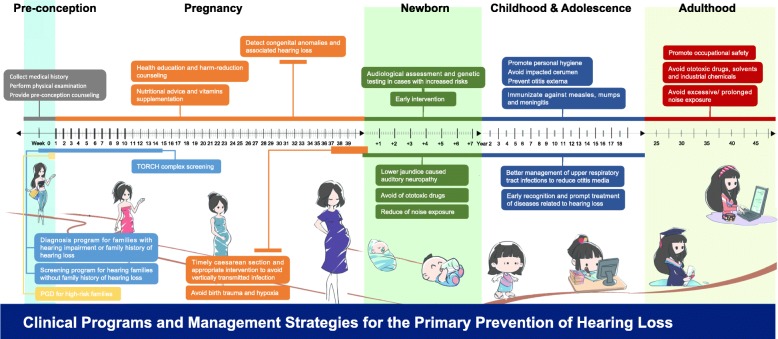
Clinical programs and management strategies in Guangdong Women and Children Hospital for the prevention of hearing loss

In the present study, we integrated the service model for the prevention and control of hearing loss with multiple maternal and child healthcare models, and developed a community-based service model to prevent hearing loss from major causes. A series of clinical programs along with an optimum timeline (Fig. 1) were adopted for the prevention and control to take place.

### Prevention and control of hearing loss in (pre-)pregnancy stage

A total of 126 couples were identified with increased genetic risks for hearing loss. After genetic counseling, 3 carrier couples chose to receive pre-implantation genetic diagnosis, 96 high-risk couples decided to proceed prenatal diagnosis, while another 27 couples chose to take audiological evaluation and genetic testing postnatally. Of them, 11 deaf couples carried biallelic mutations on the same genes would inevitablely have children with autosomal recessive hearing loss if they chose not to receive gamete donation. They were provided with detailed information and early intervention programs. Moreover, TORCH complex screening was carried out during (pre)pregnancy examination. The prevalence of serum antibodies to TORCH pathogens in the study cohort was as below. The specific IgM antibodies were found to be positive in 2.68% cases for CMV, 1.04% cases for toxoplasmosis, 1.07% cases for the Rubella virus and 0.45% for the HSV infections. The seropositvity for anti-CMV IgG (+) was 97.35%, anti-Toxoplasma IgG (+) was 25.37%, anti-HSV IgG (+) was 21.54% and anti-Rubella IgG (+) was 83.87%. Interventions for the prevention and treatment were undertaken according to the guidelines [11]. Health education and risk reduction strategies were provided through harm-reduction counseling, and the awareness rate increased from 42 to 90%. Furthermore, ultrasound tests and further pregnancy examinations detected 482 cases with structural anomalies and(or) chromosome abnormalities, proper interventions were conducted according to clinical guidelines in obstetrics and gynaecology to control important causes of congenital anomalies and associated hearing loss.

### Prevention and control of hearing loss in perinatal stage

To prevent TORCH syndrome, appropriate treatments and risk reduction strategies were employed in perinatal stage. For 7 high-risk participants with HSV lesion or prodrome presented at the time of delivery, cesarean section was recommended according to guidelines [17] to avoid vertically transmitted infection. After the study, the prevalence of TORCH syndrome halved, from 10.7 to 5.2 per 10,000. Besides these, birth trauma and hypoxia were tried to avoid by improving birth practice. For neonates with specific medical conditions, close attention and audiological assessment were provided.

### Prevention and control of hearing loss in neonatal period

A total of 24,746 newborns received UNHS, 1739 failed the initial hearing screening, and 193 of them failed the secondary screening. Those who failed both hearing screening were given audiological assessment at their third months, and 16 were diagnosed with hearing impairment. Of those, 8 were with mild hearing impairment, 6 were moderate hearing impairment and 2 were severe hearing loss. In the case of another 6023 newborns with advanced genetic risks or specific medical conditions, audiological assessment and genetic testing were provided, which identified 26 with hearing loss. Intervention programs were provided to them, and 72% received appropriate interventions initiated before 6 months of age, which was significantly ahead of the intervention time before (around 2 years old). Subsequent hearing rehabilitation through hearing aids and cochlear implants as well as suitable therapy were provided in the program. Moreover, 9 infants were indentified with deafness-associated mutations and may develop into delayed hereditary sensorineural deafness, another 7 might have genetic susceptibility to aminoglycoside ototoxicity. Detailed medication guides and timely intervention were provided to their families, with appropriate referrals and follow-ups.

### Prevention and control of hearing loss in childhood and adolescence

A student hearing healthcare program was developed for children and adolescents to promote personal hygiene and ear care, to promote immunization against illnesses related to hearing loss, and to increase their awareness about harmful factors. We gave health education in schools and communities, to promote a positive image of hearing in children and adolescents. According to questionnaire surveys before and after health education, the awareness rate of principal health knowledge about hearing healthcare increased from 37 to 86%.

### Prevention and control of hearing loss in adulthood

To get across common causes and preventive measures of hearing impairment, we provided health education through a family health care program and an occupational disease prevention program. During the study period, we gave health care consultations to more than 3000 families, provided 12 times of health education in the communities, hosted workshops for 11 times and distributed more than 8000 brochures to increase public awareness about harmful factors related to hearing impairment. According to the questionnaire survey, the awareness rate of common causes and preventive measures of hearing impairment increased to 78% in the adult respondents after the study. According to our survey, excessive noise is a major avoidable cause of hearing impairment. Thus, in adults, protection against excessive and/or prolonged noise exposure were provided. Besides, improper use of aminoglycoside antibiotics is a comparatively common cause of hearing loss in China [18]. Since mitochondrial DNA 12S rRNA gene mutations are important mechanisms of genetic susceptibility to aminoglycoside ototoxicity [19, 20], they were included in the screening tests [15], and detected 73 mutation carriers in the study cohort. Detailed medication guides were provided.

### Performance evaluation of the service model

To develop a community-based service model for the prevention and control of hearing loss, a total of 36,090 families were enrolled in the study, including 358 families with hearing impairments or family histories of hearing loss, and 35,732 hearing families without family history of hearing loss. The study lasted for 6.5 years, and 30,769 children were born during that period. A total of 42 children were born with congenital deafness, 17 of them were born into families with advanced genetic risks for hearing loss, 9 were born with specific medical conditions like prematurity, low birth weight, hyperbilirubinaemia and NICU stay, another 16 were born into general-risk families. About one third of them were diagnosed prenatally, others were diagnosed within 3 months of age, and 72% of them received interventions initiated before 6 months of age. Besides, 13 children presented with delayed hearing loss; 9 of them were diagnosed with delayed hereditary sensorineural deafness in neonatal period, and 4 were diagnosed within 3 months after onset. 1 adolescent and 5 adults suffered from hearing impairment because of accidental injuries, 2 adolescents and 7 adults appeared tinnitus, and were all diagnosed within 3 months after onset. They were detected in the ongoing surveillance system and also the self-reporting system in the program, and were provided with timely interventions as well as appropriate referrals.

Moreover, 80 families were identified with mutations in mitochondrial DNA 12S rRNA gene, which are important mechanisms of genetic susceptibility to aminoglycoside ototoxicity [19, 20]. Given the maternal inheritance, detailed medication guides were provided to the detected carriers and their maternal relatives, and may prevent aminoglycoside-induced hearing loss in approximately 800 people. Additionaly, health education and risk reduction strategies were provided for the prevention and control of common congenital infections. After the study, the prevalence of TORCH syndrome halved, from 10.7 to 5.2 per 10,000.

Furthermore, the awareness rates of principal health knowledge about hearing healthcare significantly increased through health education. In (pre)pregnant families, the awareness rate increased from 42 to 90% after receiving (pre)pregnancy counseling services. For children and adolescents, the health education have been brought to schools, and the awareness rate increased from 37 to 86%. For adults, health education were provided through occupational disease prevention system, and the awareness rate increased to 78% after the study.

## Discussion

It is suggested that half of all causes of hearing loss can be prevented through public health measures [9]. Especially in children, 60% of hearing loss is attributable to preventable causes [21]. However, due to the regional insurance policies and the public awareness towards hearing health, the national strategies for hearing healthcare are not implemented well in Guangdong and some other regions in China. Adapting national strategies for local or district projects could be an important step in implementing prevention measures, and developing community-based models could be of importance in carrying them out. In the present study, we try to develop an operable and flexible service model based on the clinical practice of maternal and child health institutes in Guangdong. In the model, a series of clinical programs along with an optimum timeline were adopted for the prevention and control of hearing loss to take place.

The service model we developed was integrated with multiple maternal and child healthcare models, including preconception care models, prenatal care and fetal monitoring models, perinatal health care models, neonatal screening models, student healthcare programs, and occupational protection programs. So that the service model for the prevention and control of hearing loss was no longer isolated or periodical, and became integrated and continuous. Given that the government input in the prevention of hearing loss was limited and the medical insurance was not satisfying, the national strategies for hearing healthcare are not implemented well in Guangdong and some other regions in China. Whereas, the maternal and child healthcare has solid foundation in China, and the service models are inclusive. Hence, we try to integrate the service model for the prevention and control of hearing loss with multiple maternal and child healthcare models, and it demonstrates with favorable performance in the clinical practice of Guangdong Province.

As important components of the model, we provided primary ear and hearing care to prevent and treat common ear conditions [22, 23], carried out early detection for deafness and hearing impairment [14, 24], made appropriate referrals and follow-ups. We implemented immunization program for vaccine-preventable diseases related to hearing loss [11]. We put efforts to improve maternal health services and to minimize hearing loss related to inadequate antenatal and perinatal care [17]. We also provided genetic counseling services to patients who have hearing impairments or family histories [14]. Public education is also an important function of maternal and child health institutes, we try to address the problem and prevention strategies through health education. However, the service model is incomplete yet. Participants might still drop out of the study because costs for detecting and treating hearing loss are mainly out-of-pocket costs. Besides, it is hard to evaluate the effects of prevention at each stage of life cycle, owing to the lack of corresponding data in the previous and the absence of adequate control. Nevertheless, the study to establish a community-based service model would be an important step to implement national strategies and to extend the coverage of hearing health services.

## Conclusions

With the world’s largest population and the new family-planning policies, hearing loss has become a significant public health concern in China. Developing community-based service models could be of importance in implementing national strategies to prevent and control hearing loss. The service model we developed was integrated with multiple maternal and child healthcare models, and demonstrated with favorable performance in the clinical practice of Guangdong Province.

## Data Availability

The datasets generated and/or analyzed during the current study are not publicly available due individual privacy but are available from the corresponding author on reasonable request.

## Abbreviations

ABR: Auditory brainstem response
UNHS: Universal newborn hearing screening
